# The role of sera from equine grass sickness on apoptosis induction in PC12 Tet-off p53 cell line

**Published:** 2015-03-15

**Authors:** Hassan Malekinejad, Nazli Alizadeh-Tabrizi, Araz Ostadi, Johanna Fink-Gremmels

**Affiliations:** 1 *Department of Basic Sciences, Faculty of Veterinary Medicine, Urmia University, Urmia, Iran; *; 2 *Department for Veterinary Pharmacology and Toxicology, Faculty of Veterinary Medicine, Utrecht University, Utrecht, The Netherlands.*

**Keywords:** Caspase-3/7, Cytochrome C, DNA fragmentation, P53 cells, PC12 Tet-off

## Abstract

The pathogenesis of equine grass sickness (EGS) has not fully understood. A better understanding of the exact pathogenesis of diseases can help to make an accurate diagnosis. Previous studies reported some pathological damage of neuronal cells in EGS patients. In this study, primarily cytotoxicity of serum from three clinically EGS-diagnosed horses on PC12 Tet-off (PTO) cells was assessed. Subsequently, the apoptotic tests including cytochrome C release, caspase-3/7 activity measurement and DNA fragmentation assay were conducted to clarify the apoptotic effect of serum from EGS patients. Addition of serum from EGS patients at concentrations higher than 25% on PTO cells resulted in a significant cytotoxicity in Alamar blue reduction assay compared with serum from healthy horses. All three apoptotic endpoints showed that the serum from EGS patients does have capability to induce apoptosis. A remarkable up regulation of cytochrome C release accompanied with concentration- and time-dependent augmentation in caspase-3/7 activity and ultimately DNA fragmentation were observed. Our data suggest that serum from EGS patients might have potentially neurotoxic compounds, which exerts cytotoxic and apoptotic effects on neuronal cells. Moreover, the EGS serum-induced apoptosis attributes to augmentation of cytochrome C release and caspase-3/7 activity.

## Introduction

Equine grass sickness (EGS) is a common and often fatal disease characterized by autonomic dysfunction of the alimentary tract. The presenting clinical signs include varying degrees of colic, intestinal stasis, anorexia, weight loss, tachycardia, excessive salivation, dysphagia, patchy sweeting, bilateral or unilateral ptosis, rhinitis sicca, and muscle tremor.^1^ EGS is associated with the selective degeneration of autonomic nervous system. The symptoms are classified as per-acute, acute, sub-acute and chronic forms. Horses with per-acute, acute and sub-acute symptoms will either die or require euthanasia within 2 days of onset, although some sub-acute cases progress to the chronic stage.^   2^

The etiology of EGS is still unknown; however, two hypotheses were investigated. One of them suggests that EGS may be caused by the bacterium *Clostridium botulinium* and the other one hypotheses that EGS is due to oxidative stress and its potential neurotoxic effects.^1^ It has also been proposed that the disease could be caused by an ingested or produced neurotoxin that is absorbed through the GI tract, severely damaging the enteric nerves. It is thought that the neurotoxin reaches the peripheral autonomic ganglia via the circulation and/or retrograde axonal transport.^3^

Histological investigations in EGS cases indicated that in gut wall plexi, prevertebral and paravertebral ganglia, the intermediolateral tract of the spinal cord and certain brain stem nuclei some neurons show a characteristics loss of stainability (chromatolysis).^4^^,^^5^ Previous studies showed that the injection of plasma from EGS cases with auto-nomic nervous system damage to normal horses resulted in a significant decrease in mitochondrial function in equine thoracic sympathetic chain ganglion cells after 24 hr.^6^^,^^7^

Apoptosis or programmed cell death can be triggered by a variety of intrinsic and extrinsic signals.^8^ It has been shown that in some cases apoptosis may be reprogrammed to the alternative type of cell death – necrosis. The crucial point in this transition from apoptosis to necrosis is suggested to be the inhibition of some proteases termed caspases, particularly, caspase-3 protease activity, which is activated in apoptotic cells but remains to be non-activate in necrotic cells.^9^^,^^10^ All caspases are expressed as pro-enzymes. Activation of caspases involves proteolytic processing. Caspases initiate and execute cell death by inactivating anti-apoptotic proteins, shutting down DNA replication and repair. 

The apoptotic executioner caspases include caspase-3, -6, and -7.^11^ It has been reported that caspases 3 and 7 do have similar function *in vitro*, highly similar three dimensional structures and also 54.0% homology in amino acid sequence.^12^ The extrinsic and intrinsic pathways are ended on the same execution pathway and this pathway is initiated by the cleavage of caspase-3 and results in DNA fragmentation, formation of apoptotic bodies, expression of ligands for phagocytic cell receptors and finally uptake by phagocytic cells.^13^ We hypothesized that apoptosis could be a possible pathway in EGS pathogenesis. To clarify the mechanism of neuropathy of EGS in *in vitro* cultured genetically engineered PC12 Tet-off (PTO p53) cells, which infected by p53 protein, the key targets in apoptosis pathway including mitochondrial function, cytochrome C release, caspases-3/7 activation and finally DNA fragmentation were examined.

## Materials and Methods

**Chemicals. **Tetracyclin, hygromycin B, diaminocyclo-hexane-N, N, N’, N’ tetra-acetic acid, were purchased from Sigma Chemical Co. (St. Louis, USA). Alamar blue (AB) was purchased from Biosource International (Biosource, The Netherlands). RPMI 1640 cell culture medium; geneticin (G418), Horse serum, Tet-off fetal bovine serum (FBS), and trypsine EDTA, were applied by Gibco Life Technology Ltd. (Paisley, UK). Apo-one TM homogenous caspase-3/7 assay kit was obtained from Promega, Corp. (Madison, USA). The other chemicals were purchased from Sigma Chemical Co. (St. Louis, USA). Serum samples of clinically diagnosed EGS patients were obtained from the Centre for Equine Medicine, Faculty of Veterinary Medicine, Utrecht University, Utrecht, The Netherlands. Serum samples were collected from three stallions between March and August 2012. The pooled serum samples from clinically healthy horses (n = 3) were used as control for each part of study. 

PC12-p53 cells were a kind gift from Dr. Silvia Stingele, European Centre for the Validation of Alternative Methods, Milan, Italy.

**Cell Culture. **PTO-p53 cells were grown in collagen Vitogen-100 coated tissue culture flasks in RPMI-1640 supplemented with 10% horse serum, 5% Tet Off-FBS, 1% L-glutamine, 150 µg mL^-1 ^G418, 150 µg mL^-1 ^hygromycin B, 2 µg mL^-1 ^tetracycline and 1% penicillin (100 IU mL^-1^), streptomycin (100 µg mL^-1^). Cells were incubated at 37 ˚C in a humidified atmosphere of 5% CO_2_ in air. 

**Cell treatment.** PTO-p53 cells were harvested from stuck culture and were plated in 96- and 6- well culture collagen-coated plates at density of 20,000 cell per well 0.2 mL supplemented RPMI 1640 medium without tetracycline for AB reduction and caspase-3/7 activity measurement and 1.6 × 10^6^ cells per well in 4 mL supplemented RPMI-1640 medium without tetracycline for cytochrome C release and DNA-laddering experiments. Cells were incubated for 48 hr at 37 ˚C in a humidified atmosphere of 5% CO_2_ in air. After 48 hr incubation (37 ˚C and 5% CO_2_), the medium was removed and replaced with fresh medium containing EGS serum or control serum at different concentrations. Following 24 hr treatment, AB reduction as an endpoint for mitochondrial activity and apoptotic effects of EGS sera were assessed. 

**Cytotoxicity study with Alamar blue reduction assays.** Mitochondrial activities was measured following treatment with EGS serum by using AB reduction assay according to Bull *et al.*^14^ Briefly, the commercially bought stock solution of AB was diluted 10-fold in RPMI-1640. Following 24 hr incubation of the cells with the medium containing pooled serum sample from three clinically diagnosed EGS cases and/or with the pooled serum sample from clinically healthy horses, the medium was removed and the cells washed with warmed PBS. Medium containing AB (Diluted, 1:10) and cells incubated for 3 hr at 37 ˚C. 

The fluorescence of the medium, due to the reduced AB, was measured by an excitation wavelength of 560 nm and an emission wavelength of 590 nm (Cytoflour 2300; Millipore, Bedford, USA). Cell viability was expressed as:

$\mathrm{Cell viability}=\frac{\mathrm{Fluorescence value of treated cells}}{\mathrm{Fluorescence value of control cells}}\times 100$

**Cytochrome C assay. **Following given treatment period cells were harvested with buffer containing 0.25 M sucrose, 0.1 mM ethylenediamine tetra-acetic acid, and 1 mM phenylmethanesulfonyl fluoride, and kept in ice for 15 min. Cells were disrupted using a glass homogenizer according to previously described method.^15^ Following centrifugation at 14000 *g* for 15 min, 5 mg of cytosolic protein was fractionated by SDS-PAGE analyze by Western blot using a monoclonal rat antibody against cytochrome C. Detection was done by rinsing of monoclonal rat Anti-cytochrome C (Bioscience) against mouse (IgG2b) for immunological staining. Protein concentrations of the PTO-p53 cells were determined based on Lowry *et al*.^16^ Densitometric analyses of Western blots were performed using Molecular analyst software (Version 1.5; BioRad, Hercules, USA). 

**Caspase-3/7** **activity** **assessment**. Following 4, 8 and 18 hr treatment of PTO-p53 cells with 100 μL of different concentrations of EGS sera, 100 μL of homogeneous caspase-3/7 reagent was added on cells and cultured plates covered with aluminum foil and gently mixed contents of wells using a plate shaker at 300 to 500 rpm for 30 sec to 1 min. Cells incubated for 3 hr and the fluorescence of each single well was measured at an excitation wavelength of 498 nm and emission wavelength of 530 nm. Caspase-3/7 activity were expressed as below:

Caspase-3/7 activity=Fluorescence value of treated cellsFluorescence value of control cells×100

**DNA-Laddering. **DNA of treated PTO-p53 cells was purified based on apoptotic DNA Laddering kit manufacturer’s instructions (Roche Diagnostics GmbH, Mannheim, Germany). Briefly, cultured medium was removed and the treated cells washed once with pre-warmed PBS and the cells were harvested by lyses buffer (6 M guanidine-HCL, 10 mM Urea, 10 mM Tris-HCL, 20% Triton X-100 [v/v], pH 4.4) and incubated for 10 min at 15 to 25 ˚C. After which time isopropanol was added to the samples. The filter and collection tubes (provided in the kit) were combined and samples pipetted into the upper reservoir. After centrifugation for 1 min at 8,000 rpm (Eppendorf Centrifuge 5417 R) the flow through was discarded and the used collection tube were combined with filter. A volume of 500 μL washing buffer (20 mM NaCl; 2 mM Tris-HCL; 80% [v/v] ethanol, pH 7.5) was added to the upper reservoir, centrifuged at 8,000 rpm for 1 min, and after discarding the collection tube this washing phase was repeated and the residual wash buffer was removed by centrifuging for 10 sec at 13,000 rpm. Then filter tube was inserted in a clean eppendorf tube and for the elution of DNA, 200 μL of 72 ˚C pre-warmed elution buffer (10 mM Tris, pH 8.5) was added to the filter tube and centrifuged for 1 min at 8,000 rpm. The eluted DNA was used directly or stored at – 20 ˚C for subsequent analysis. 

DNA was quantified and a volume of elute corresponding to 2 µg DNA (15-17 μL of eluted DNA) was added to loading buffer (50% glycerol; 2mM EDTA; 0.4% bromphenol blue), and the DNA solution was run on a 0.8% agarose gel for 60 min at 60 V constant voltage. PST1 also was loaded as a marker for identification of amount of DNA. Gels were stained with ethidium bromide and visualized by Gel Doc 2000 system (Bio-Rad). 

**Statistical analysis. **All numerical values are expressed as a percentage of controls, cells treated with serum obtained from healthy horses, set at 100%. Significant differences between control and EGS patients were determined by one-way ANOVA followed by Bonferroni post hoc test using GraphPad Prism (Version 4.0; GraphPad software Inc., San Diego, USA). Values were considered to be significant when *p* < 0.05.

## Results

**Fig. 1:**
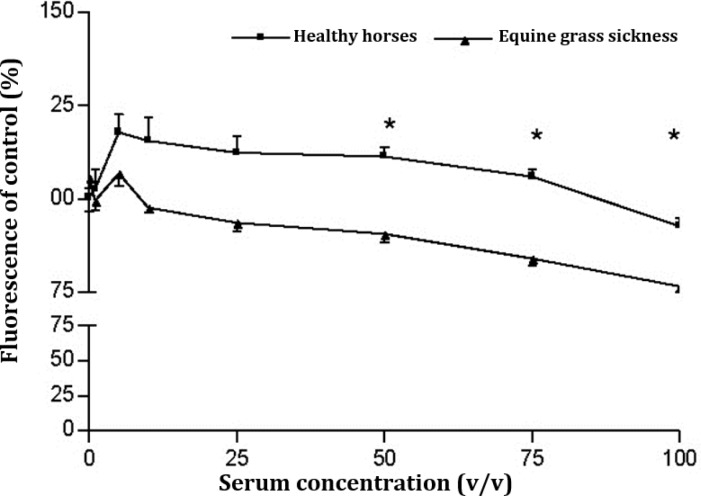
Effect of pooled sera from EGS (n = 3) and healthy horses (n = 3) on AB-reduction in PTO cells; PTO cells were exposed against various concentrations of sera for 24 hr and the cell viability was assessed by using the AB-reduction assay. Stars are indicating a significant (*p* < 0.05) difference between healthy and EGS sera.

**Alamar blue reduction. **Results of AB reduction by EGS sera in PTO-p53 cells indicated that EGS serum from clinically EGS diagnosed horses affected the function of mitochondria and cell viability assay showed a concentration-dependent toxicity. Significantly difference (*p* <0.001) appeared between sera of healthy horses as the control and sera from EGS patients at concentrations higher than 25.0% (v/v). Also it became clear that serum from healthy horses at all given concentrations resulted in a cell proliferation on PTO-p53 cells (Fig. 1). 

**Fig. 2:**
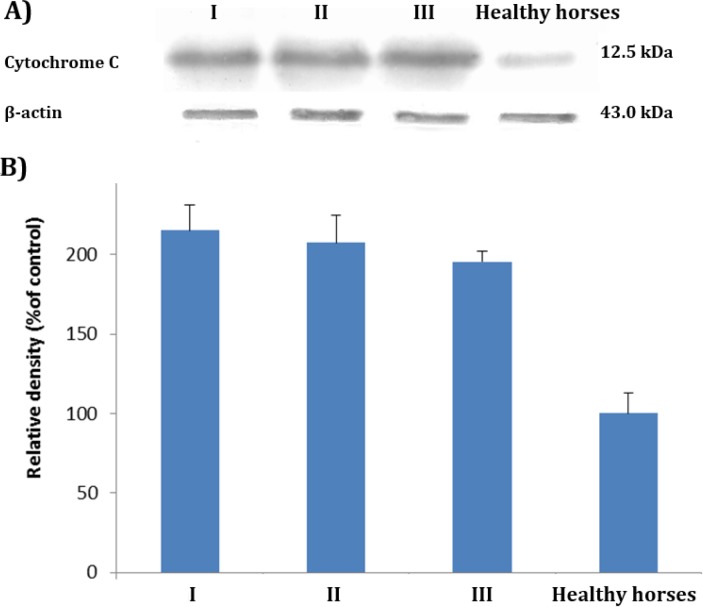
Cytochrome C release following treatment with EGS sera; **A)** shows the cytochrome C release from treated cells with sera from EGS and healthy horses and **B)** demonstrates the densito-metric analysis of released amount of cytochrome C, which normalized based on the corresponding β -actin bands.

**Cytochrome C release. **Cytochrome C release examination after exposing the PTO-p53 cells for 24 hr against sera from EGS cases and the pooled serum from clinically healthy horses at 50 % (v/v) concentration, showed that all three EGS sera could up-regulate the cytochrome C release in comparison to control serum (Fig. 2). The expression of cytochrome C (12.5 kDa) in EGS or healthy horses sera treated PTO cells was normalized based on the expression of β-actin (43.0 kDa). The densitometry analyses which obtained based on three times repeat, represent the quantitative elevation of the cytochrome C release in cells which exposed to EGS sera compared to control group. 

* indicates a significant difference (*p *< 0.05) among the amount of released cytochrome C from cells exposed to sera from healthy horses and sera from EGS patients.

**Fig. 3:**
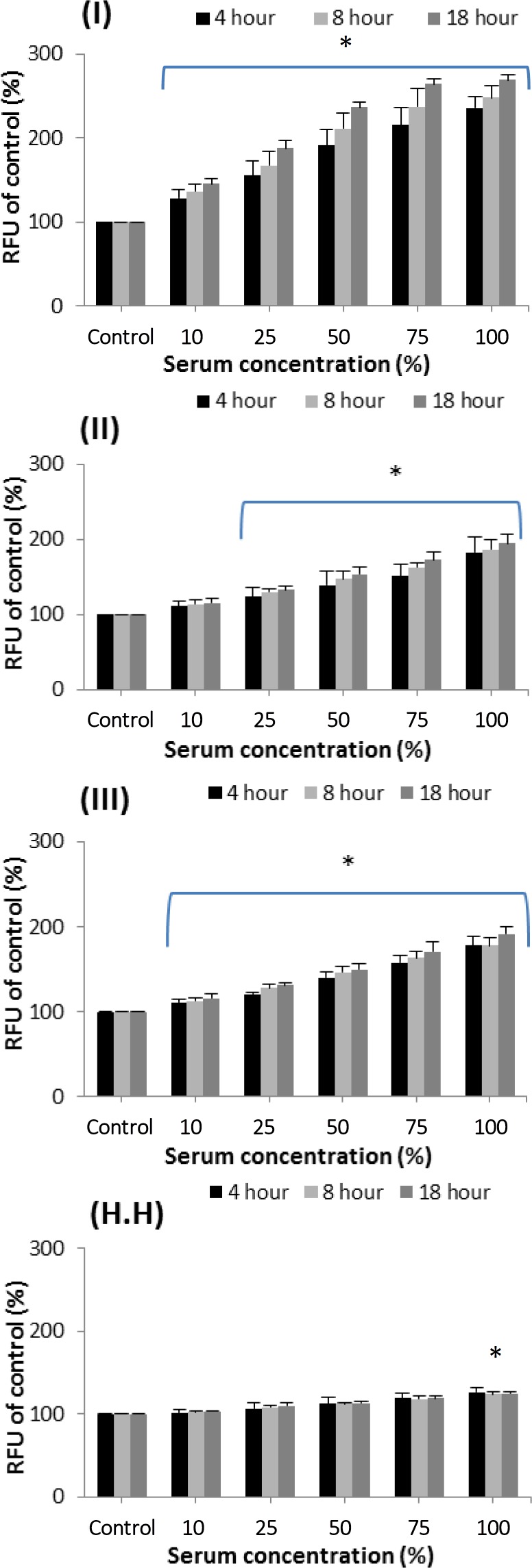
Effect of EGS serum on caspase-3/7 activity in PTO cells; Cells were exposed for various time periods (4, 8, and 18 hr) against sera from either EGS cases (I, II and III panels) or pooled sera from three healthy hoses (H.H) and the activity of caspase-3/7 were measured. All the columns represent the mean of triplicate values and error bars indicate the standard deviation. RFU: Relative fluorescence units.

**Caspase-3/7 activity. **Caspase-3/7 activities in exposed PTO-p53 cells against EGS sera were measured at 3 time points. EGS serum from all three horses elevated the caspase-3/7 activities in a concentration- and time-dependent fashion (Figs. 3A, 3B and 3C). The elevation of caspase-3/7 activities was found remarkably profound in case No. I than other two cases. Incubation of PTO-p53 cells against the pooled serum from healthy horses showed no significant (*p* > 0.05) alteration in caspase-3/7 activities (Fig. 3D). 

* indicates a significant difference (*p* < 0.05) among control and other treated cells at all time periods of 4, 8 and 18 hr.

**Fig.4:**
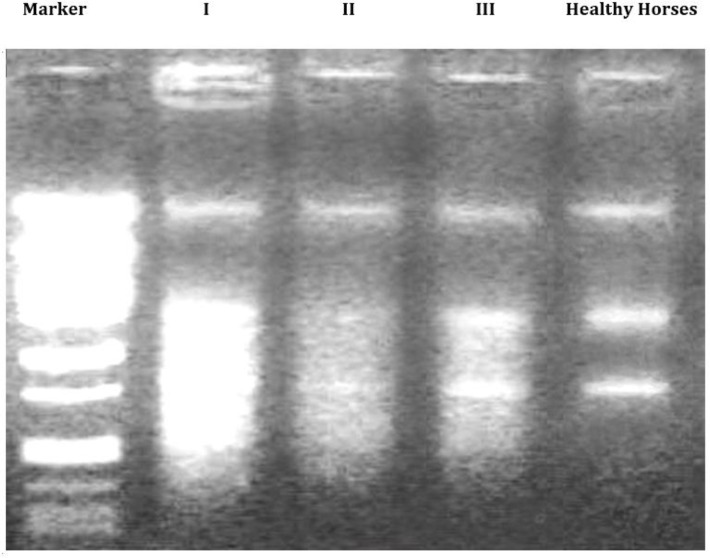
DNA-fragmentation by EGS serum; the lanes 2, 3, and 4 indicate the DNA fragmentation due to exposing the PTO cells against sera from EGS horses of No. I, II, and III and the lane 5 represents the effect of sera from three healthy horses on PTO cells, respectively. Lane 1 represents a ladder marker.

**DNA-Laddering. **DNA fragmentation has occurred after exposing PTO-p53 cells for 24 hr against EGS sera at 50.0% (v/v) concentration (Fig. 4). Slightly, differences were observed between three EGS cases; with sever DNA laddering in lane II belonging to horse No. I and followed by lane 3 and 4 as representative for horses No. II and III. No smear form of DNA was found in the cells which exposed to serum from healthy horses.

## Discussion

The results of present study demonstrated the cytotoxic and apoptotic effects of serum from EGS horses on PTO-p53 cells. Our results also showed that PTO-p53 cells as a genetically engineered cell line could be useful diagnostic tool for EGS patients. PC12 Tet-off cell line is stably transformed with protein Tet-off and therefore expresses the tetracycline-controlled transactivator (tTA) in a stable way.^18^ P53 gene located on chromosome 17q codes for predominantly nuclear 53.0 kDa phospho-protein, p53. The p53 is expressed at very low levels in normal cells. It accumulates in response to DNA damaging agents such as UV-irradiation or γ–irradiation and genotoxic compounds, or physiological stress conditions, such as hypoxia and heat. The central role of p53 in mediating cell death in response to a vide array of stimuli such as DNA damaging agents and growth factor with-drawal and its capacity to induce spontaneous cell death in some cultured cell lines after overexpression has been well documented.^17^ Therefore, selecting the PTO-p53 cells as highly sensitive cells to DNA damaging agents for this study was to highlight easily the cytotoxic and apoptotic effects of serum from clinically diagnosed EGS cases.

Alamar blue reduction following exposing to EGS sera showed the mitochondrial function reduced remarkably in PTO-p53 cells in a concentration-dependent fashion indicating that the higher concentration of EGS, the higher toxicity. Mitochondria are important cell apparatuses and related with cell breathing, oxygen metabolism, enzyme activity and energy supply.^19^^-^^21^All of those functions related to the permeability of the mitochondria and mitochondrial transmembrane potential (MTP). When MTP decreases, the mitochondria generate morphological and functional changes.^22^ Also, it is thought that the early phase of both modes of cell death may involve similar changes in mitochondrial membrane permeability.^23^^-^^25^ Our results are supported with other researchers as Marchenko *et al*. showed that a fraction of p53 protein elevated in mitochondria at the onset of p53-dependent apoptosis.^26^ The accumulation of p53 in mitochondria is rapid and precedes changes in mitochondrial membrane potential, cytochrome C release, and procaspase-3 activation. Yuan *et al*. presented that p53-induced apoptosis involves early lysosomal destabilization and latter mitochondrial damage, including the decrease of membrane potential and release of cytochrome C.^27^ Principally, quickly after mitochondrial permeability changing, due to release of cytochrome C or apoptosis-inducing factor (AIF) some specific cysteine proteases namely caspases that cleave after aspartic acid residues are activated. Caspases catalyze a highly selective pattern of protein degradation.^28^ Also, it was found that mitochondrial products are necessary for the induction of apoptosis in nuclei of mammalian cells. ^29^^,^^30^

Cytochrome C by itself is not apoptogenic, but AIF can stimulate the proteolytic activation of caspase-3. The results of cytochrome C release in this study showed that there are indeed differences between control and affected EGS sera on PTO p53 cells. Increasing of cytochrome C release after exposing the cells against all three selected horse sera, indicating of highly likely involvement of this pathway (mitochondria changes–cytochrome C release enhancing -caspase-3/7 activity increase) in PTO cell death and in turn confirming prior occurred events in mitochondria. This hypothesis was confirmed in the current study with caspase-3/7 activity measurement and it became clear that in the all three studied cases of EGS, the activity of caspase-3/7 significantly increased, although there are differences between three studied cases.

Our DNA-laddering experiment results indicated that serum from horse No. I very severely and sera from horses No. II and III moderately, caused the DNA fragmentation in exposed cells. These results are in line with AB reduction, cytochrome C release and caspase-3/7 activity assays. Thus, again DNA fragmentation results suggest that apoptosis may be the main way of EGS neuropathy. Previous studies about the pathogenesis of EGS indicated that many neurons are lost in EGS by apoptosis, which is characterized by cell shrinkage, chromatin aggregation with extensive genomic fragmentation and nuclear pyknosis.^31^

According to our findings in this study it may be suggested that the sera from EGS horses likely contain unusual and at the same time toxic compound(s), which could cause cytotoxic and apoptotic effects. Previous data are in accordance with our suggestion as it has been shown that an unusual compound that was related to neurotoxin present in the original serum of horses with acute cases of EGS, and that substance has not been detected in the serum of normal horses or cases of colic and chronic cases of EGS.^32^ Some later studies also support our suggestion that EGS is caused by neurotoxin which can gain access to the circulation, and may selectively destroy autonomic neurons by retrograde axonal transport.^33^

Differences between studied cases may be explained by four-overlap clinically and obviously para-clinically forms of disease. Thus, it might be concluded that the cases, which showed profound amelioration of cytochrome C release, caspase-3/7 activity and DNA fragmentation, they could be categorized in pre-acute or acute stages of disease. There are very early studies showing the presence of various amounts of neurotoxin(s) in circulation of EGS affected horses between different stages of disease and equally important the absence of such compounds in the serum of healthy horses.^34^

In conclusion, our results highlighted that exposing of PTO-p53 cells against EGS serum results in a significant mitochondrial toxicity, elevating the cyto-chrome C release which in turn results in enhancing of caspase-3/7 activity and ultimately DNA fragmentation. Moreover, all tested endpoints especially caspase-3/7 activity measurement are appropriate tests and PTO cells are suitable cells for *in vitro* para-clinically pre-mortem examination diagnosis of EGS. However to enhance the sensitivity of the *in vitro* tests and to uncover the exact pathogenesis of the EGS, further studies with high sample size are needed.
